# Cortical activity during cognitive and walking tasks in individuals with chronic nonspecific low back pain: a functional near-infrared spectroscopy study

**DOI:** 10.3389/fnins.2026.1778661

**Published:** 2026-04-23

**Authors:** Fan Jiang, Haiting Huang, Yifeng Chen, Xin Li, Jinjin Wei, Jiatao Wen, Pubu Wang, Yaobin Long, Chongwu Xiao, Guozhi Huang

**Affiliations:** 1 Center for Rehabilitation Medicine, Zhujiang Hospital, Southern Medical University, Guangzhou, China; 2 Department of Rehabilitation Medicine, The First Affiliated Hospital of Guangxi Medical University, Nanning, China; 3 Department of Rehabilitation Medicine, The Second Affiliated Hospital of Guangxi Medical University, Guangxi Medical University, Nanning, China; 4 The Second Clinical Medical College of Guangxi Medical University, Guangxi Medical University, Nanning, China

**Keywords:** chronic nonspecific back pain, dual task, functional near-infrared spectroscopy, gait, neuroimage

## Abstract

**Introduction:**

Previous research demonstrates that individuals with chronic nonspecific low back pain (CNSLBP) exhibit changes of gait patterns. However, the neural mechanisms responsible for these adverse events remain unelucidated. In this study, we used functional near-infrared spectroscopy (fNIRS) to investigate cortical activities during cognitive and walking tasks to provide evidence of the central mechanisms responsible for changes of gait patterns in individuals with CNSLBP.

**Methods:**

In this cross-sectional study, we evaluated 18 individuals with CNSLBP (the CNSLBP group) and 18 healthy controls (the HC group) under three specific conditions: Task 1 (a single walking task), Task 2 (a single cognitive task) and Task 3 (a cognitive-walking dual task). Cortical activities were measured using fNIRS, including the bilateral premotor cortex and supplementary motor area (PMC/SMA), primary motor cortex (M1), somatosensory association cortex (SAC), and primary somatosensory cortex (S1). Gait parameters, including step duration, step length, stride length, velocity, cadence, swing power, and cycle, were measured using a three-dimensional gait analysis system.

**Results:**

In Task 1, the CNSLBP group exhibited a significantly lower velocity (*p* = 0.029) and higher activation in the left SAC (*p* = 0.001) and right S1 (*p* = 0.018) than that of the HC group. In Task 2, the CNSLBP group exhibited higher activation in the left SAC (*p* = 0.028), right SAC (*p* = 0.033), and left S1 (*p* = 0.032). In Task 3, the CNSLBP group exhibited significantly lower step length (*p* = 0.031), stride length (*p* = 0.041), velocity (*p* = 0.016), and swing power (*p* = 0.047). Correlation analysis in Task 1 revealed stronger associations between parameters in the CNSLBP group.

**Conclusion:**

Our findings suggest that individuals with CNSLBP exhibit distinct patterns of cortical activities and gait performance. The SAC and S1 were involved in walking, and central sensitization was observed in individuals with CNSLBP in daily cognitive and walking tasks. These findings could contribute to the recovery and rehabilitation of CNSLBP.

## 1 Introduction

Chronic nonspecific low back pain (CNSLBP) is defined as pain located between the lower rib margin and the gluteal folds, persisting for more than 3 months; however, without an identifiable specific underlying cause (Dionne et al., 2008; Koes et al., 2006). The prevalence of CNSLBP is progressively increasing on an annual basis, comprising approximately 85% of all low back pain cases (Verbrugghe et al., 2020; Maher et al., 2017). Characterized by high incidence and high disability rates, CNSLBP has become a widespread global health problem and imposes a substantial economic burden on society, including healthcare costs, compensation, productivity loss, and employee retraining (Thelin et al., 2008). As pressures on individuals continue to increase in terms of both work and study, the CNSLBP population is becoming progressively younger, and the societal burden is likely to rise in the future (Swain et al., 2014; Hestbaek et al., 2006).

The etiology of CNSLBP is complex. Evidence suggests that changes in the structure and function of the lumbosacral muscle tissues, which mainly include reduced strength in the lumbar disc musculature accompanied by adipose tissue infiltration, may play a key role in its development (Ozkan et al., 2025; Teichtahl et al., 2015). Considering a normal gait usually relies on neuromuscular regulation and close coordination of the lower limb joints (Rum et al., 2021), the changes of lumbosacral muscle tissues may impair gait. Gait is a complex motor function, which refers to the manner or pattern of walking, and is influenced by a variety of physical and neurological factors. Gait abnormalities often manifest as alterations in step duration, step length, velocity, stride, cadence, and cycle, which can result from disruptions in the ability of central nervous system to process sensory and motor information (Minassian et al., 2017). In addition, studies have highlighted the relationship between CNSLBP and gait patterns, suggesting a potential bidirectional interaction in which each condition may exacerbate the other (Bonab et al., 2020; Pocovi et al., 2024; Haddadj et al., 2025). Pain intensity was negatively correlated with stride length, step length, cadence, and velocity (Bonab et al., 2020). Walking training could significantly reduce the risk of occurrence and recurrence of low back pain (Pocovi et al., 2024; Haddadj et al., 2025). Exploring the gait patterns in individuals with CNSLBP, and further investigating the central mechanisms underlying these patterns, are crucial if we are to develop targeted rehabilitation strategies to address pain management and issues with dynamic stability.

Three-dimensional gait analysis is an advanced gait analysis technique that can quantitatively and accurately assess gait parameters during walking, including joint kinematics and spatiotemporal characteristics (Ito et al., 2022). Previous studies on gait in individuals with chronic low back pain have reported mixed findings, with some research highlighting changes in walking patterns such as slower speed or reduced stride length (Barzilay et al., 2016; Elbaz et al., 2009), whereas others have failed to identify consistent alterations (Rum et al., 2021; Lin et al., 2023). These inconsistent results suggest that the nature of gait disturbances in this population warrants further investigation. Moreover, it is essential to recognize that gait is not merely a reflection of peripheral musculoskeletal function but is simultaneously regulated by central neural pathways (Wurdeman et al., 2012; Gimmon et al., 2017). Previous study identified a specific correlation between gait parameters and activation of the dorsolateral prefrontal cortex in individuals with chronic low back pain (Nguyen et al., 2023). However, traditional gait measurement systems, which often rely on static or peripheral data collection methods, have constrained our understanding of the central neural mechanisms involved in changes of gait patterns in individuals with CNSLBP. Furthermore, considering the prevalence of motor-cognitive dual tasks in the daily lives of individuals with CNSLBP, an investigation of hemodynamic responses during walking under dual task conditions could provide additional insights into the central mechanisms that contribute to altered gait patterns in this population.

Functional near-infrared spectroscopy (fNIRS) is a non-invasive neuroimaging technique that measures cortical activation by detecting changes in oxygenated hemoglobin (HbO) levels in response to neural activity (Liu et al., 2025). Unlike traditional devices that focus primarily on static data, fNIRS allows for the real-time monitoring of cortical activation during dynamic tasks, including walking. Furthermore, fNIRS offers more advantages compared to traditional neuroimaging devices, including portability, ease of use, and the ability to measure cortical activity in natural environments, making it an ideal tool for studying complex tasks involving cognitive and motor functions (McDonald and Perdue, 2018). Despite its potential, studies using fNIRS to examine the neural mechanisms of gait in individuals with CNSLBP, remain limited.

Therefore, the aim of this study was to use fNIRS and three-dimensional gait analysis to investigate gait performance and cortical activation during cognitive and walking tasks in individuals with CNSLBP. Based on our current understanding of CNSLBP, we hypothesized that individuals with CNSLBP would exhibit alterations in gait pattern and cortical activation during cognitive and walking tasks when compared to healthy controls. Understanding the specific cortical activities involved would provide valuable insights into the central mechanisms contributing to the changes of gait patterns, offering a more comprehensive understanding of CNSLBP and its impact on motor function.

## 2 Materials and methods

### 2.1 Participants

This was a cross-sectional study. From August to December 2025, participants were recruited from the Department of Rehabilitation at the Second Affiliated Hospital of Guangxi Medical University and nearby communities. Finally, a total of 18 individuals with CNSLBP (the CNSLBP group) and 18 healthy controls (the HC group) were included. The study was approved by the Ethics Committee of the Second Affiliated Hospital of Guangxi Medical University (approval number: 2025-KY (0376), approved on 18 March 2025). All participants provided written informed consent in accordance with the Declaration of Helsinki (Goodyear et al., 2007).

Sample size was calculated using G*Power version 3.1.9.2 (Kiel University, Kiel, Germany). Based on the results of a preliminary pilot study, the concentration of HbO in the left somatosensory association cortex (SAC) during the single walking task was 0.0141 mmol/L*mm and −0.0092 mmol/L*mm for five individuals with CNSLBP and five healthy controls, respectively, with standard deviations (SDs) of 0.0201 and 0.0226. The calculated effect size was 1.09. With an α level set at 0.05, a statistical power of 0.8, and a 1:1 allocation ratio between the two groups, the required sample size was determined to be 30 participants, with at least 15 participants in each group.

The inclusion criteria for the study were as follows: (a) diagnosis of CNSLBP according to international diagnostic standards (Chou et al., 2007); (b) right-handed; (c) aged between 18 and 75 years, with a visual analog scale (VAS) score ≥4 cm on a 0–10 cm scale; (d) an ability to comply with the study protocol and sign an informed consent form; and (e) no treatment for low back pain administered over the last month. The exclusion criteria were as follows: (a) imaging results indicating specific pathologies causing low back pain (e.g., spinal tuberculosis, vertebral fractures, infections, tumors); (b) the presence of other diseases that affect walking ability (e.g., knee osteoarthritis, lower limb fractures); (c) severe cardiovascular diseases and neurological disorders (d) a history of back surgery; (e) the presence of other pain conditions besides low back pain (e.g., neck pain, headache); and (f) pregnancy.

### 2.2 Clinical measurements

Demographic data were primarily collected by self-reporting from the participants, including age, sex, height, weight, body mass index (BMI), years of education, and duration of pain. Pain intensity in the CNSLBP group was assessed using the VAS. A straight line (10 cm in length) was drawn on a piece of paper, with 0 cm indicating no pain and 10 cm representing severe pain. Participants marked the point on the line that corresponded to their pain level, with higher values indicating more severe pain (Thong et al., 2018). The degree of functional impairment in the CNSLBP group was assessed using the Roland–Morris Disability Questionnaire (RMDQ), which includes 24 items related to dressing, daily activities, walking, standing, and sleeping. The total RMDQ score ranges from 0 to 24, with higher scores indicating severe functional impairment (Fan et al., 2012). The level of catastrophizing pain in the CNSLBP group was assessed using the Pain Catastrophizing Scale (PCS), which reflects the psychological impact of pain and includes three dimensions: rumination, magnification, and helplessness. The total PCS score ranges from 0 to 52, with higher scores indicating more severe pain catastrophizing (Majumder et al., 2020; Xiao et al., 2025). We also used the Short Form 12 Health Survey (SF-12) to assess the life quality of all participants, covering two major dimensions: physical health and mental health. Higher SF-12 scores indicate a better life quality (Lin et al., 2020). All of these measurements were acquired prior to the cognitive and walking tasks, taking approximately 30 min.

### 2.3 Gait measurements

Gait parameters were acquired by an Intelligent Device for Energy Expenditure and Activity (IDEEA) (MiniSun LLC, Fresno, California, United States). This device is a wearable gait acquisition system based on a miniature three-dimensional accelerometer. The system consists of one main unit and five three-dimensional accelerometers. Miniature accelerometer sensors were attached to the bilateral thighs, feet, and sternum of the participants to collect gait parameters during walking. Data were wirelessly transmitted in real-time to the main unit located at the waist, with a sampling frequency of 64 Hz. The IDEEA system has demonstrated good reliability and has been validated in previous studies on gait performance measurement (Marsh et al., 2007). Based on prior studies (Chen et al., 2022; Li et al., 2023), the gait parameters collected in this study included step duration, step length, stride length, velocity, cadence, swing power, and cycle. Following the acquisition of the raw data, it was uploaded from the main unit to the computer acquisition software. The gait waveform diagrams corresponding to the timed walking tasks were identified and subsequently labeled. The system autonomously averaged the gait parameters over the duration of the tasks and produced comprehensive gait analysis results in a spreadsheet format, which could be readily utilized for further analysis.

### 2.4 fNIRS measurements and data processing

**Figure 1:**
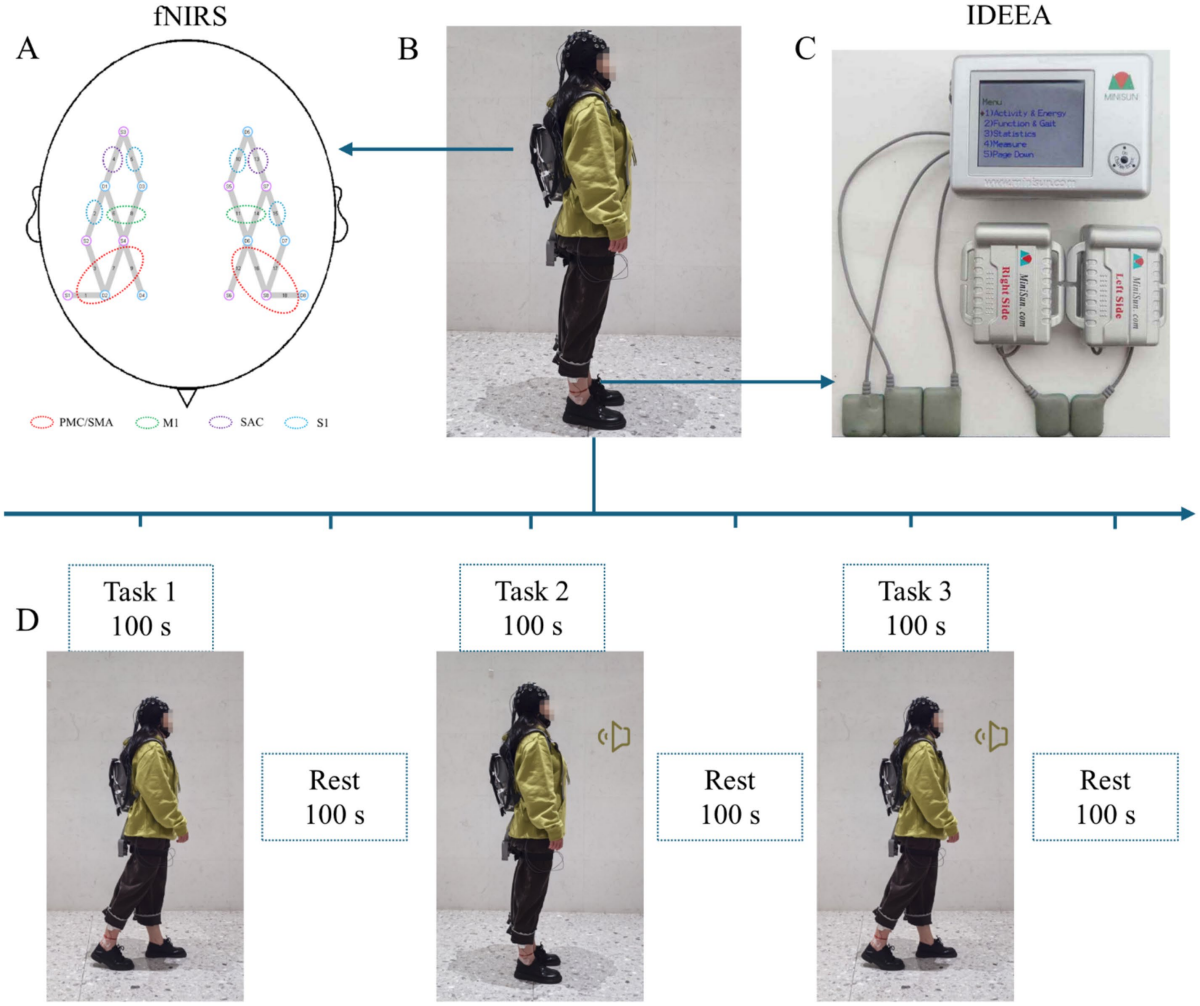
Experimental design. **(A)** The channel configuration diagram of the brain regions of interest for fNIRS. **(B)** Schematic diagram of the experimental preparation. **(C)** Schematic diagram showing configuration of the IDEEA device. **(D)** Schematic diagram of three cognitive and walking tasks. The horn symbol indicates the need to perform a cognitive task. The masked person displayed in the figure agreed to the use of the anonymized image in this publication.

A portable 18-channel fNIRS device NirSmart-3000DS (Danyang Huichuang Medical Equipment Co., Ltd., Danyang, China) was used to record cortical activation. The system consists of near-infrared light sources and avalanche photodiodes as detectors. The wave lengths of the light source probes were 730 nm and 850 nm, with a sampling rate of 11 Hz. The experiment employed eight light source probes and eight detection probes, forming 18 effective channels. The average distance between the light emitter and detector was 3 cm. The system was positioned according to the international 10/20 system, and the obtained coordinates were then converted to Montreal Neurological Institute (MNI) coordinates. The spatial registration method in NirSpace (Danyang Huichuang Medical Equipment Co., Ltd., Danyang, China) was further applied to project the data onto the standard MNI brain template. Based on this anatomical localization and previous studies (Li et al., 2023; Yan et al., 2024), we identified several specific regions of interest (ROIs) for analysis: the bilateral premotor cortex and supplementary motor area (PMC/SMA), primary motor cortex (M1), SAC, and primary somatosensory cortex (S1). The left PMC/SMA (L-PMC/SMA) was composed of channels 12, 16, 17, and 18, while the right PMC/SMA (R-PMC/SMA) was composed of channels 1, 3, 7, and 9. The left M1 (L-M1) consisted of channels 11 and 14, while the right M1 (R-M1) consisted of channels 6 and 8. The left and right SAC (L-SAC and R-SAC) was covered by channels 13 and 4, respectively. The left S1 (L-S1) consisted of channels 10 and 15, while the right S1 (R-S1) consisted of channels 2 and 5 (Figure 1A). Group analysis was performed based on these ROIs. In this study, we focused on HbO concentration as a marker of cortical activity, as this is considered to represent the most sensitive and reliable indicator of changes in regional brain oxygenation (Suzuki et al., 2008; Xiao et al., 2025).

Following acquisition of the raw fNIRS data, we performed further analysis with Nirspark software (Danyang Huichuang Medical Equipment Co., Ltd., Danyang, China). The process primarily consisted of the following steps: (a) converting the raw optical intensity data into optical density data; (b) removing motion artifacts by spline interpolation; (c) using a bandpass filter between 0.01 and 0.1 Hz to remove physiological noise (e.g., respiratory, cardiac activity, and low frequency signal drift); (d) converting the denoised optical density data into hemoglobin concentration data; and (e) using the modified Beer–Lambert law to calculated the relative concentration changes in HbO, with the differential path length factor set to 6 for each wavelength. Baseline correction was applied to the HbO concentration for the first 5 s before the cognitive and walking tasks, and changes in HbO concentration relative to this baseline were calculated during each task to reflect cortical activation (Xu et al., 2024). Block-averaged results were generated by aggregating and averaging the HbO concentration within each block.

### 2.5 Experiment procedures

Prior to the experiment, we ensured that the surrounding environment was quiet and well-lit. Participants were instructed to wear the fNIRS and IDEEA devices in the required manner and stand in a corridor approximately 50 m in length (Figures 1B,C). Participants were then informed that they would perform three cognitive and walking tasks of varying difficulty: (a) Task 1, a single walking task; (b) Task 2, a single cognitive task; and (c) Task 3, a cognitive-walking dual task. For the walking task, participants were instructed to walk at a comfortable pace to closely mimic their daily walking patterns. To obtain a complete gait waveform for each task in the IDEEA device without segmentation due to stopping, participants were instructed to walk along both sides of the corridor and follow a bigger arch-shaped route when turning. This approach was necessitated by the corridor’s limited length and was implemented to maximize data accuracy. For the cognitive task, participants would hear a series of numbers broadcast every 10 s, with a range of three to five digits. After each broadcast, participants were required to rapidly recall the numbers in reverse order. Prior to the experiment, a therapist who was unaware of pain status guided the participants through practice sessions to ensure an accuracy rate above 70%. The order of the tasks was kept confidential from the participants, who were simply instructed to follow voice guidance throughout the experiment. Baseline data were collected by fNIRS during a resting state for 10 s. Subsequently, Tasks 1, 2, and 3 were performed for 100 s each, with a 100-s rest period between each task to allow the HbO concentration to return to baseline levels (Figure 1D). If a participant stopped, fell, or had an accuracy rate too low when recalling numbers during the task, the measurement would be terminated. Before attempting the task again, participants were given a 10-min rest period.

### 2.6 Statistical analysis

Data analysis was performed using SPSS software version 27.0 (IBM Corp., Armonk, NY, United States). First, normality (Shapiro–Wilk test) and homogeneity of variance (Levene’s test) were tested to assess whether data met the assumptions for parametric analysis. Categorical variables are presented as frequency (percentage) whereas continuous variables are presented as means (with SD). For demographic data, Chi-squared tests were used to compare sex differences between the two groups, and independent sample *t*-tests were used for all other comparisons. When the data were normally distributed, independent sample *t*-tests were used to compare differences in cortical activation and gait parameters between the two groups during the cognitive and walking tasks, and the effect size was further calculated using Cohen’s d (d). Otherwise, non-parametric tests were considered. For correlation analysis, Pearson’s correlation analysis was used to investigate the relationship between HbO concentration and gait parameters when the data were normally distributed. Otherwise, Spearman’s correlation analysis was applied. GraphPad Prism version 10.0 (GraphPad Software, San Diego, CA, United States) was used for graph and chart editing. *p* < 0.05 was considered a statistically significant difference.

## 3 Results

### 3.1 Demographic characteristics

A total of 36 participants were included in this study (18 in each group). As shown in Table 1, there were no statistically significant differences between the two groups in terms of age, sex, height, weight, BMI, and years of education (*p* > 0.05). For the CNSLBP group, the mean duration of pain was 50.94 months, the mean VAS score was 6.14 cm, the mean RMDQ score was 9.44 points, and the mean PCS score was 30.33 points. The mean SF-12 score for the HC group was 40.11 points, which compared to 29.50 points in the CNSLBP group (*p* < 0.001).

**Table 1:** Demographical and clinical data for the two groups.

Variable	CNSLBP group	HC group	*p* value
Age, y	45.67 (15.46)	37.67 (14.78)	0.122
Sex, f/m	5 (28%)/13(72%)	8 (44%)/10 (56%)	0.298
Height, m	1.60 (0.08)	1.62 (0.06)	0.521
Weight, kg	56.28 (9.62)	61.69 (10.22)	0.111
BMI, kg/m^2^	22.01 (3.62)	23.66 (3.79)	0.192
Education, y	12.06 (5.17)	13.28 (4.43)	0.452
Duration, month	50.94 (57.96)	–	–
VAS, cm	6.14 (1.01)	–	–
RMDQ, point	9.44 (3.7)	–	–
PCS, point	30.33 (7.23)	–	–
SF-12, point	29.50 (4.37)	40.11 (2.27)	<0.001

Scores are presented as mean (SD). CNSLBP, chronic nonspecific low back pain; HC, healthy control; y, years; f, females; m, males; BMI, Body Mass Index; VAS, visual analog scale; RMDQ, Roland–Morris Disability Questionnaire; PCS, Pain Catastrophizing Scale; SF-12, Short Form 12 Health Survey.

### 3.2 A comparison of gait performance during cognitive and walking tasks between the two groups

Statistical analysis (Table 2) revealed that during Task 1, the CNSLBP group was significantly slower than the HC group in terms of velocity (*p* = 0.029, *d* = 0.759), with no significant differences between the two groups for other gait parameters (*p* > 0.05). During Task 3, the CNSLBP group exhibited a significantly lower step length (*p* = 0.031, *d* = 0.750), stride length (*p* = 0.041, *d* = 0.706), velocity (*p* = 0.016, *d* = 0.847), and swing power (*p* = 0.047, *d* = 0.691) than the HC group. There were no significant differences between the two groups in terms of the remaining gait parameters (*p* > 0.05).

**Table 2:** A comparison of gait parameters during recognitive and walking tasks between groups [mean (SD)].

Variable	CNSLBP group (*n* = 18)	HC group (*n* = 18)	*p* value	Cohen’s d
Task 1				
Step duration (s)	0.644 (0.075)	0.604 (0.052)	0.068	0.627
Step length (m)	0.539 (0.039)	0.562 (0.033)	0.070	0.625
Stride length (m)	1.079 (0.082)	1.123 (0.070)	0.093	0.576
Velocity (m/s)	0.858 (0.125)	0.943 (0.098)	**0.029**	0.759
Cadence (steps/min)	94.977 (10.725)	100.581 (8.207)	0.087	0.587
Swing power (G)	0.396 (0.161)	0.489 (0.152)	0.081	0.600
Cycle (s)	1.287 (0.152)	1.206 (0.100)	0.067	0.634
Task 3				
Step duration (s)	0.682 (0.059)	0.643 (0.094)	0.139	0.504
Step length (m)	0.518 (0.041)	0.549 (0.041)	**0.031**	0.750
Stride length (m)	1.036 (0.086)	1.096 (0.084)	**0.041**	0.706
Velocity (m/s)	0.777 (0.093)	0.877 (0.138)	**0.016**	0.847
Cadence (steps/min)	89.434 (7.599)	95.329 (12.041)	0.088	0.586
Swing power (G)	0.327 (0.115)	0.432 (0.18)	**0.047**	0.691
Cycle (s)	1.361 (0.116)	1.286 (0.186)	0.159	0.480

Significant differences are shown in bold. Data are presented as mean (SD). CNSLBP, chronic nonspecific low back pain; HC, healthy control; Task 1: single walking task; Task 3: cognitive-walking dual task.

### 3.3 A comparison of cortical activation during cognitive and walking tasks between the two groups

**Figure 2:**
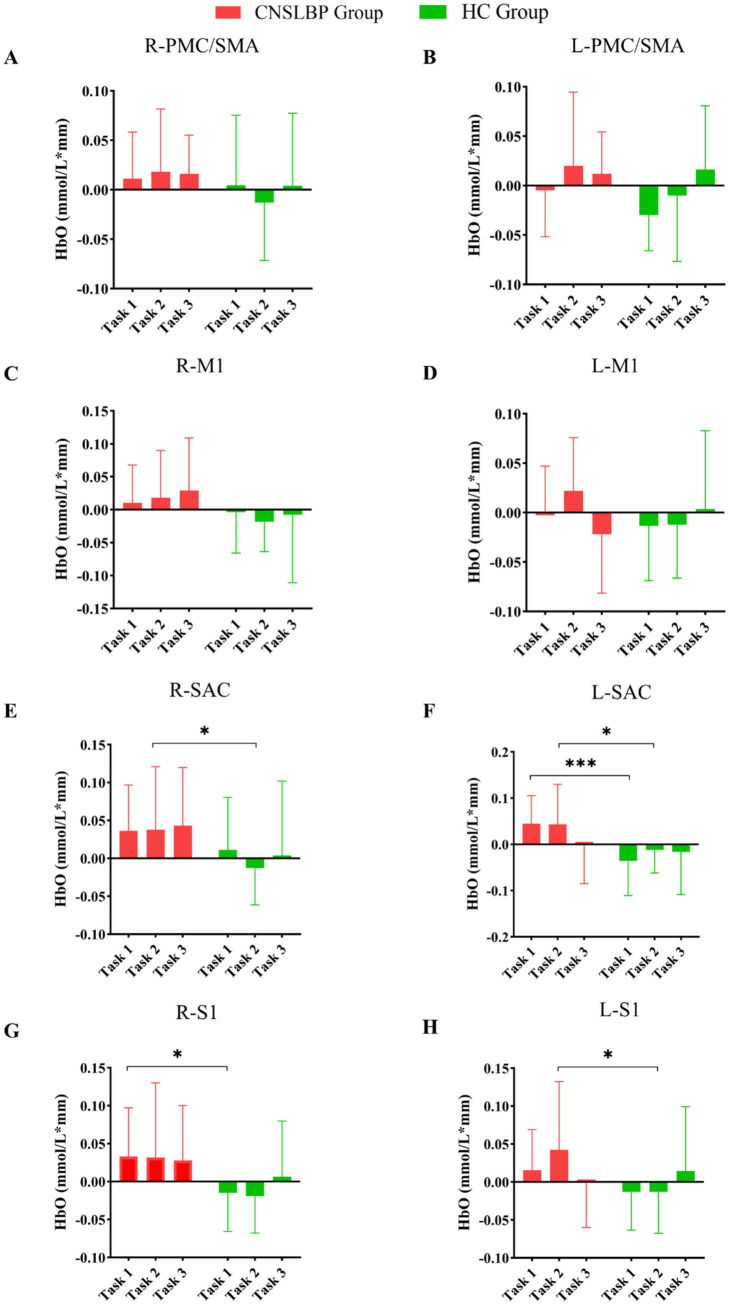
Cortical activation between the CNSLBP and HC groups in **(A)** R-PMC/SMA, **(B)** L-PMC/SMA, **(C)** R-M1, **(D)** L-M1, **(E)** R-SAC, **(F)** L-SAC, **(G)** R-S1, and **(H)** L-S1 when performing Task 1 (a single walking task), Task 2 (a single cognitive task), and Task 3 (a cognitive-walking dual task). L: left; R: right; PMC/SMA: premotor cortex and supplementary motor area; M1: primary motor cortex; SAC: somatosensory association cortex; S1: primary somatosensory cortex. *p < 0.05; ***p < 0.001.

**Figure 3:**
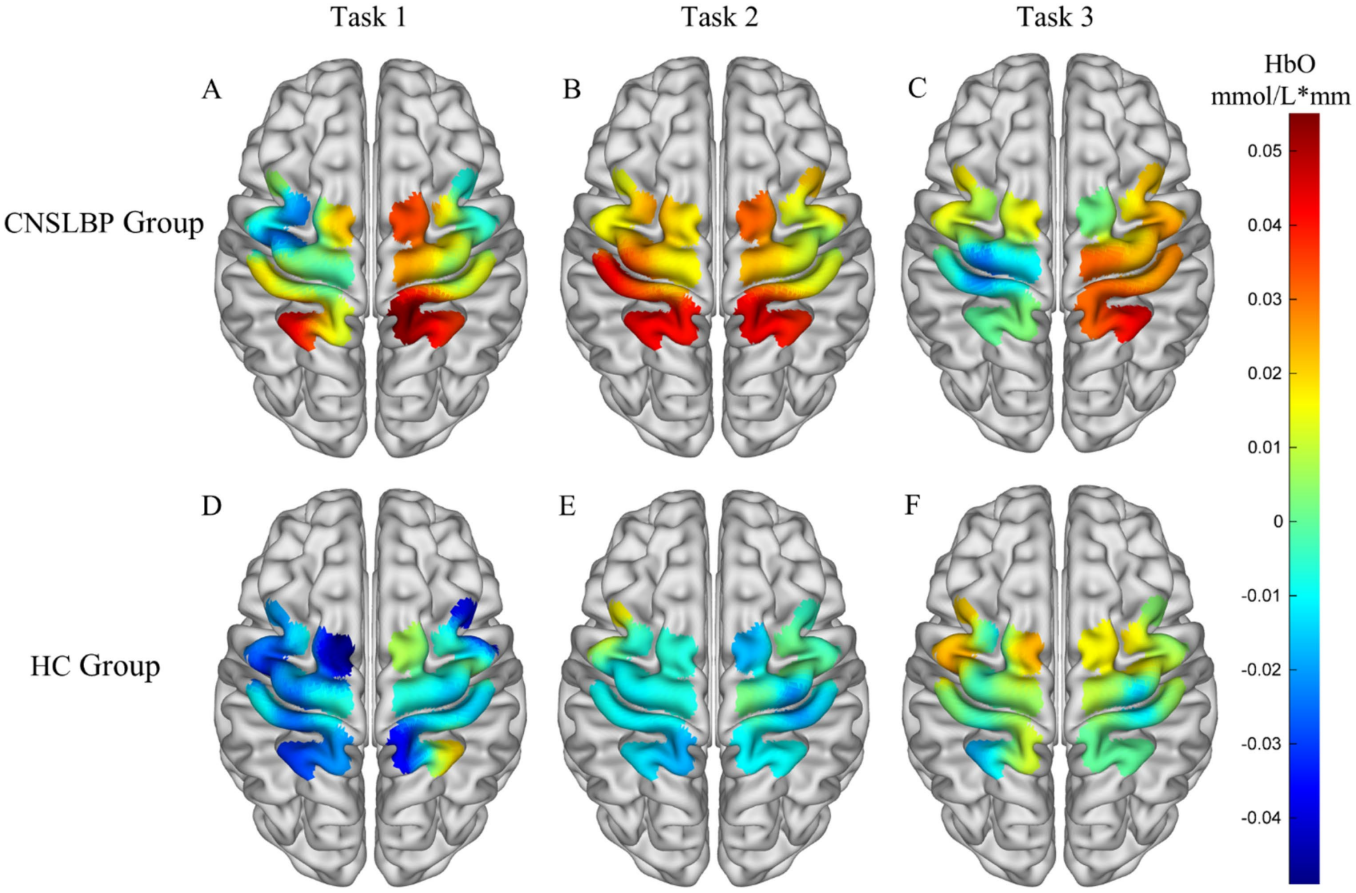
Three-dimensional (3D) brain map of mean cortical activation in tasks **(A)** 1, **(B)** 2, and **(C)** 3 in the CNSLBP group and tasks **(D)** 1, **(E)** 2, and **(F)** 3 in the HC group. Red and blue represent hyperactivation and hypoactivation, respectively.

Statistical analysis revealed that during Task 1, the CNSLBP group exhibited significantly greater activation in the L-SAC (*p* = 0.001, *d* = 1.190) and R-S1 (*p* = 0.018, *d* = 0.832) than for the HC group. There were no significant differences between the two groups in terms of the activation of other ROIs. During Task 2, the CNSLBP group exhibited significantly greater activation in the L-SAC (*p* = 0.028, *d* = 0.775), R-SAC (*p* = 0.033, *d* = 0.742), and L-S1 (*p* = 0.032, *d* = 0.744) when compared to the HC group. During Task 3, there were no significant differences in the activation of any ROI between the two groups (Figure 2). Figure 3 depicts mean cortical activation in both groups during the three tasks.

### 3.4 Association between oxygenated hemoglobin in the cerebral cortex and gait parameters

**Figure 4:**
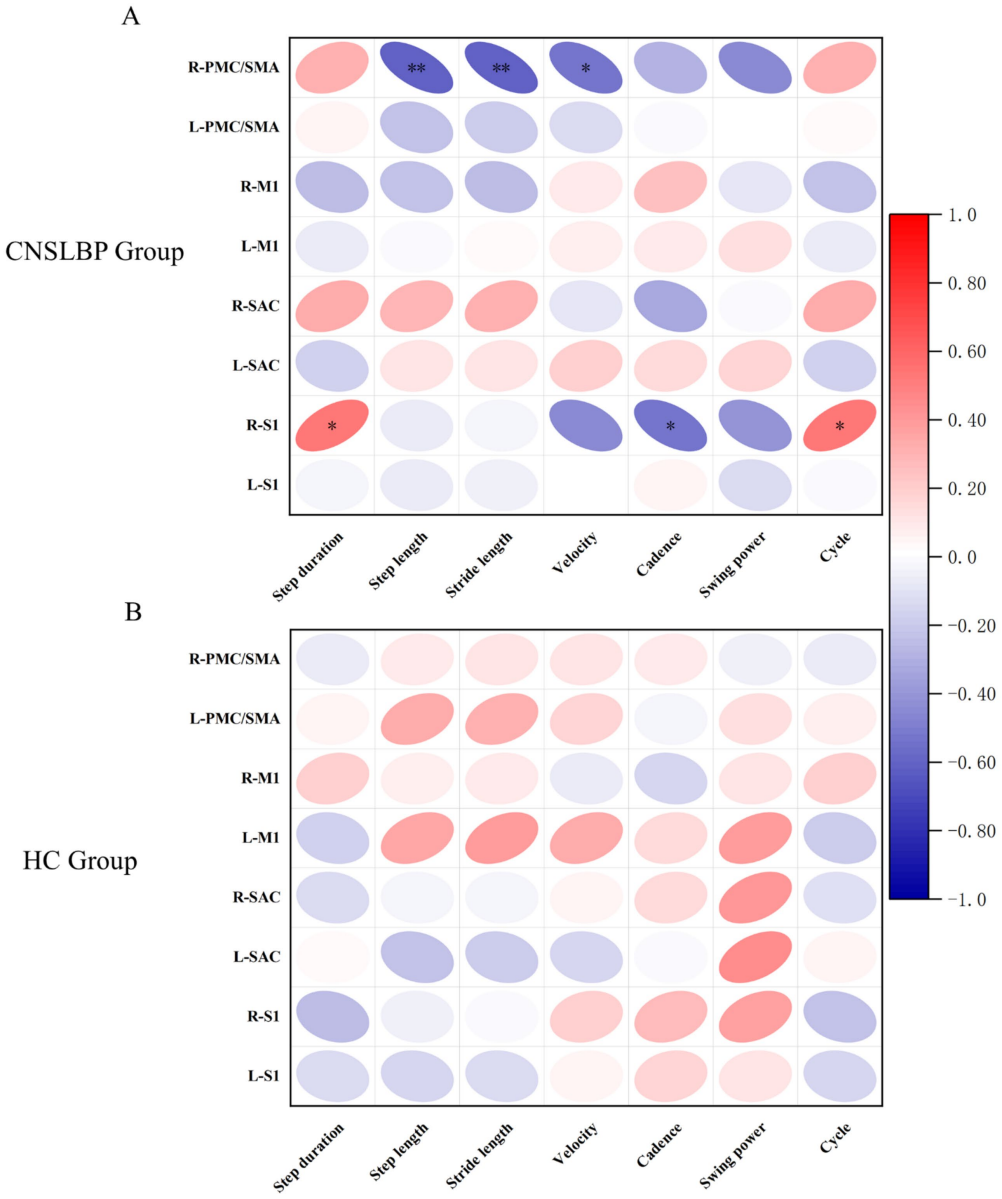
Heat map showing the correlation between cortical HbO concentration and gait parameters in the **(A)** CNSLBP and **(B)** HC groups during Task 1. L: Left; R: Right; PMC/SMA: pre-motor cortex and supplementary motor area; M1: primary motor cortex; SAC: somatosensory association cortex; S1: primary somatosensory cortex. The red color represents a positive correlation, while the blue color represents a negative correlation. *p < 0.05; **p < 0.01.

Correlation analysis revealed that during Task 1, the HbO concentration in R-PMC/SMA of the CNSLBP group was significantly and negatively correlated with step length (*p* = 0.007, *r* = −0.612), stride length (*p* = 0.007, *r* = −0.608), and velocity (*p* = 0.024, *r* = −0.528). Furthermore, HbO concentration in R-S1 was significantly and positively correlated with step duration (*p* = 0.021, *r* = 0.539) and cycle (*p* = 0.022, *r* = 0.536), and significantly and negatively correlated with cadence (*p* = 0.023, *r* = −0.532) (Figure 4A). For the HC group, no correlations were observed between HbO concentrations and gait parameters (*p* > 0.05) (Figure 4B).

## 4 Discussion

Previous studies have indicated that individuals with CNSLBP may exhibit changes of gait patterns. To explore the cortical hemodynamic activation patterns associated with these changes, we investigated gait performance and cortical activation during three different cognitive and walking tasks and further explored the relationship between these two factors. Significant differences in cortical activation were detected between the two groups during the single walking and single cognitive tasks. In the cognitive-walking dual task, the CNSLBP group performed worse in terms of gait parameters when compared to the single walking task. Furthermore, a correlation between cortical activation and gait parameters was detected in the CNSLBP group, and the correlation was clearly weaker in the HC group.

In this study, we observed significant differences in gait parameters between the two groups in both the relatively simple Task 1 and the more demanding Task 3. In Task 1, the CNSLBP group demonstrated a significantly lower walking speed than the HC group, with a moderate effect size of 0.759, which mean a clinically significant difference. A previous study reported that individuals with chronic low back pain exhibited a slower walking speed, a longer step duration, and greater step length variability, and noted that these changes may have been associated with fear-avoidance behaviors (Bailes et al., 2025). Moreover, previous studies reported that after a certain course of biomechanical treatment, individuals with CNSLBP exhibited significant improvements in terms of velocity, step length, and cadence, and that these changes were accompanied by a reduction in pain intensity (Barzilay et al., 2016; Elbaz et al., 2009). These findings further indicated that gait velocity in individuals with CNSLBP differed from that in healthy individuals, and that this parameter could serve as a useful indicator for evaluating treatment efficacy. The possible reason for the observed changes in gait pattern for individuals with CNSLBP is that these individuals are known to exhibit localized spinal kinematic changes during walking and greater variability in trunk motion, especially in the transverse plane (Rum et al., 2021; Christe et al., 2017). These kinematic and neuromuscular changes may influence gait velocity. Furthermore, in Task 3, participants in the CNSLBP group not only exhibited a slower velocity than the HC group, they also exhibited a lower step length, stride length, and swing power, with differences showing moderate to large effect sizes, presenting clinically meaningful differences between the two groups. A previous study suggested that gait pattern in individuals with chronic low back pain may be influenced by environmental factors, with gait control changes observed in laboratory settings potentially being more pronounced in the daily life environment (Nishi et al., 2021). Furthermore, these changes were also related to various factors such as central sensitization, distraction of attention, and motor control strategies (Homs et al., 2022). Compared to the HC group, the CNSLBP group had to manage walking and cognitive tasks in Task 3, and cope with additional low back pain stimulation (6.14 cm for a mean VAS score). These complex conditions may contribute to further differentiation in gait pattern. A reduction in velocity, step length, stride length, and swing power leaded to decreased impact upon foot-ground contact and diminished biomechanical forces transmitted along the lower limb to the lumbar region, thereby mitigating the exacerbation of pain. Overall, our findings were consistent with previous studies (Barzilay et al., 2016; Elbaz et al., 2009), indicating that individuals with CNSLBP exhibited changes of gait patterns in their daily life, and that these differences were more pronounced under the cognitive-walking dual task condition. Future research should employ gait performance across various tasks as objective outcome measures to more thoroughly assess the efficacy of rehabilitation for CNSLBP.

The portability of fNIRS allows this tool to be used in dynamic experiments, such as monitoring cortical activities during movement or social interactions; this is difficult to achieve with traditional neuroimaging techniques. In the present study, activation of the L-SAC and R-S1 in the CNSLBP group during Task 1 was significantly higher than that of the HC group, with effect sizes of 1.190 and 0.832, respectively. Effect sizes exceeding 0.8 represent large, clinically meaningful differences detectable in the patient assessments. Although there were no significant differences in the activation of other ROIs, the CNSLBP group consistently exhibited higher activation levels. A previous study showed that individuals with CNSLBP experienced central sensitization, leading to the sustained amplification and disinhibition of pain signals. The core feature of this condition was an increased excitability of nociceptive pathways and a reduction in inhibitory regulatory functions (Schuttert et al., 2021). Consistent with the previous study, the CNSLBP group in our study may also develop central sensitization due to prolonged chronic pain stimulation. Specifically, the cortical excitability was abnormally increased during the simple Task 1, with the most pronounced activation observed in the L-SAC and R-S1. The primary function of the S1 is to precisely localize and quantify body sensations, while the function of the SAC is predominantly related to bilateral sensory comparison and the integration of pain and emotional memory. Differences in the activation in these two sensory cortical areas indirectly reflected the impact of pain on the gait of individuals with CNSLBP. In summary, due to the influence of pain, individuals with CNSLBP must engage more of the sensory cortex, limiting resources for walking control and altering gait patterns. Future research should explore central interventions targeting the sensory cortex to assess the effectiveness in improving CNSLBP rehabilitation outcomes.

In Task 2, the CNSLBP group exhibited significant activation in both the bilateral SAC and L-S1, with activation levels higher than those observed in Task 1. The central sensitization within the sensory cortex may indicate the activation of a compensatory mechanism in individuals with CNSLBP. Due to the limited capacity of central resources, the prefrontal cortex, which is responsible for working memory, was inadequate to sustain the execution of Task 2 under the influence of pain. Consequently, additional cortical regions were recruited to preserve working memory functionality. Moreover, a previous study reported a significant association between cognitive-behavioral factors and central sensitization symptoms in individuals with CNSLBP (Huysmans et al., 2018). The pattern of cortical activation observed indicated that the cognitive load in our study was sufficiently high to further induce central sensitization in individuals with CNSLBP. Furthermore, central sensitization has also been highly correlated with certain psychological factors such as depression and catastrophizing pain (Dahmani et al., 2023). The CNSLBP participants in our present study exhibited clear catastrophizing pain which may have also contributed to the observed differences in cortical activation. In cases where chronic pain endures, individuals with CNSLBP often cultivate catastrophic cognitions regarding severe injury, which in turn activate the affective circuit involving the anterior cingulate cortex, insula, and amygdala. This activation results in a diminished function of the descending inhibitory system, ultimately leading to heightened excitability of dorsal horn neurons within the spinal cord and the subsequent development of central sensitization (Sluka and Clauw, 2016). Further correlation analysis of our study revealed a significant positive link between HbO concentrations in L-SAC (*p* = 0.027, *r* = 0.519) and L-S1 (*p* = 0.037, *r* = 0.495) and PCS scores during Task 2 in the CNSLBP group (details in Supplementary material), which was consistent with the theory.

In Task 3, there was no significant difference between the two groups in terms of cortical activation. Compared to Tasks 1 and 2, the CNSLBP group generally exhibited a low activation pattern in Task 3, while the HC group generally showed a pattern of high activation. One possible reason underlying these findings is that the CNSLBP group may have experienced an increased cognitive and motor resource load during the dual task, while also being disrupted by chronic pain. This demand in resources could have exceeded the threshold, leading to central protective inhibition (Rietschel et al., 2012). In contrast, individuals in the HC group, who were free from pain, could allocate more cognitive and motor resources to the dual task, thus resulting in higher levels of activation. The cross-domain competition model of dual task suggested that there was a limitation in the total amount of resources available for allocation in the human body. When multiple tasks are performed within a certain time frame, postural control and cognitive activities compete for resources. Consequently, the capacity allocated to each task decreased, leading to a decline in task performance (Lacour et al., 2008; Xiao et al., 2023). Consistent with this hypothesis, we observed more pronounced changes of gait patterns and lower cortical activation in the CNSLBP group during Task 3.

During Task 1, significant differences were detected between the two groups in both cortical activation and gait performance. Therefore, we conducted further correlation analyses which showed that activation in the R-PMC/SMA and R-S1 was significantly correlated with gait parameters in the CNSLBP group, but not in the HC group. One possible explanation is that the central sensitization and resource reallocation strategy adopted by the CNSLBP group required gait performance to rely on the sustained involvement of the PMC/SMA and S1, thereby leading to enhanced gait-central coupling. In contrast, gait control in the HC group was highly automated and did not require the engagement of an additional neural resource (Zheng et al., 2022). A previous study investigated the associations between gait parameters and prefrontal activation during walking in individuals with chronic low back pain and reported small-to-moderate correlations between gait parameters and activation in the dorsolateral prefrontal cortex (Nguyen et al., 2023). However, the ROIs in this study were limited to the prefrontal cortex. In contrast, in our study, we focused on the sensorimotor cortex and detected statistically significant differences, thus enhancing the existing literature. In summary, multivariate correlation analysis between cortical activation and gait parameters in the CNSLBP group suggested the presence of neural compensatory responses (Wang et al., 2016). Individuals with CNSLBP compensated for impaired sensorimotor function by activating the right PMC/SMA and S1.

This study has several limitations that need to be considered. First, due to equipment constraints, we only measured cortical activation in the sensorimotor cortex during the cognitive and walking tasks; we did not investigate activation in the prefrontal cortex. Future studies should focus on a broader range of ROIs. Second, during gait analysis, we selected integrated gait parameters from both sides. Conducting unilateral gait analysis would facilitate our understanding of gait asymmetry in individuals with CNSLBP, providing a deeper insight into changes in gait pattern.

## 5 Conclusion

Consistent with previous studies, individuals with CNSLBP were found to exhibit changes of gait patterns. fNIRS data from both groups revealed that the SAC and S1 were involved in the daily walking task, specifically indicated by increased activation. Further correlation analysis revealed neural compensatory effects during cognitive and walking tasks in individuals with CNSLBP. These findings extend our understanding of cortical activity, gait performance, and their relationship in individuals with CNSLBP during cognitive and walking tasks. Our findings have begun to elucidate the neural mechanisms of gait pattern changes in individuals with CNSLBP. Future studies should investigate whether changes in cortical activity in individuals with CNSLBP improve following strategic rehabilitation.

## Data Availability

The original contributions presented in the study are included in the article/Supplementary material, further inquiries can be directed to the corresponding authors.
