# Supplementary material for: Cortical activity during cognitive and walking tasks in individuals with chronic nonspecific low back pain: a functional near-infrared spectroscopy study
Source: Front Neurosci. 2026 Apr 23;20:1778661. doi: 10.3389/fnins.2026.1778661 (PMC13149265; doi:10.3389/fnins.2026.1778661)
Supplement: Supplementary file 1 [file Table_1.docx]

**Supplementary Table 1.** Correlation analysis between HbO concentration and PCS score in the CNSLBP group during single cognitive task.

|  |  | R-PMC/SMA | L-PMC/SMA | R-M1 | L-M1 | R-SAC | L-SAC | R-S1 | L-S1 |
| --- | --- | --- | --- | --- | --- | --- | --- | --- | --- |
| PCS | r | 0.368 | -0.029 | 0.335 | 0.390 | 0.321 | 0.519 | 0.385 | 0.495 |
|  | *p* | 0.133 | 0.908 | 0.174 | 0.109 | 0.194 | **0.027** | 0.114 | **0.037** |

L: left; R: right; PMC/SMA: pre-motor cortex and supplementary motor area; M1: primary motor cortex; SAC: somatosensory association cortex; S1: primary somatosensory cortex; PCS: Pain Catastrophizing Scale.
